# AAV-norrin gene therapy rescues retinal defects in mice with Norrie disease and oxygen-induced retinopathy

**DOI:** 10.1016/j.omta.2026.201795

**Published:** 2026-06-24

**Authors:** Andrea E. Dillinger, Herbert Jaegle, Holger Fuchs, Benjamin Strobel, Norbert Redemann, Ernst R. Tamm

**Affiliations:** 1 Institute of Human Anatomy and Embryology, University of Regensburg, Regensburg, Germany; 2 Institute of Neuroanatomy, University Clinic Hamburg-Eppendorf, Hamburg, Germany; 3 Department of Ophthalmology, University Clinic of Regensburg, Regensburg, Germany; 4 Boehringer Ingelheim Pharma GmbH & Co. KG, Biberach an der Riss, Germany

**Keywords:** retinal angiogenesis, retinopathy of prematurity, Wnt signaling, AAV-mediated gene therapy, blood-retinal barrier, ocular gene delivery

## Abstract

Norrin, secreted by retinal Müller cells, activates canonical Wnt signaling via Frizzled-4 and co-receptors. Loss-of-function mutations abolish intraretinal capillary formation in mice. In humans, mutations in *NDP*, which encodes norrin, cause Norrie disease, characterized by retinal hypovascularization and congenital blindness, and X-linked familial exudative vitreoretinopathy (FEVR), resembling retinopathy of prematurity (ROP). We evaluated adeno-associated viral (AAV) vectors expressing norrin as gene therapy for Norrie disease, FEVR, and ROP. AAV2-7m8 and AAV-ShH10 were tested in juvenile wild-type and norrin-deficient (*Ndp*^KO^) mice via intravitreal injection at postnatal day 7, with some mice subjected to oxygen-induced retinopathy (OIR). AAV2-7m8 transduced Müller glia, while AAV-ShH10 targeted retinal ganglion cells. Both vectors fully restored intraretinal capillary growth in *Ndp*^KO^ mice, normalizing vessel density and plexus organization, preserving the blood-retinal barrier, and rescuing visual function. In OIR, scAAV2-7m8-huNorrin reduced vaso-obliteration and neovascular tuft formation, increasing deep plexus coverage, and suppressed *Vegfa164* and *Ang*-2 upregulation. The findings demonstrate that AAV-mediated norrin delivery efficiently targets retinal glia and neurons, restores vascular structure and function, stabilizes the blood-retinal barrier, and mitigates OIR-induced pathological angiogenesis, supporting its potential as a therapeutic strategy for Norrie-related retinopathies and ROP.

## Graphical abstract

**Figure:**
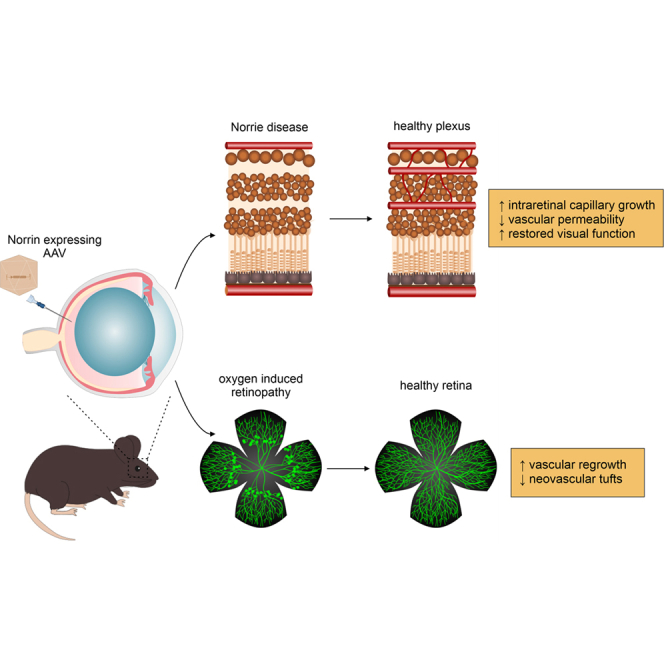

## Teaser

Dillinger and colleagues demonstrated that AAV-mediated norrin delivery to the retina restores intraretinal vascular architecture, preserves the blood-retinal barrier, and rescues visual function in Norrin-deficient mice, while also suppressing pathological angiogenesis in oxygen-induced retinopathy. These findings support AAV-Norrin gene therapy as a promising strategy for Norrie disease, FEVR, and ROP.

## Introduction

Norrin is a small signaling molecule predominantly secreted by retinal Müller cells, the principal glial cells of the retina.^1^^,^^2^^,^^3^ It belongs to the cysteine-knot growth factor superfamily and functions as a ligand for the canonical Wnt signaling pathway via the endothelial receptor Frizzled-4 (FZ4).^4^ Norrin/FZ4 signaling is essential for the development of mature retinal capillaries.^5^^,^^6^^,^^7^^,^^8^ Mice carrying loss-of-function mutations in the genes coding for norrin, FZ4, or the co-receptors low-density lipoprotein receptor-related protein-5 (LRP5) and tetraspanin-12 (TSPAN) lack intraretinal capillaries.^7^^,^^9^ Mutations in the human *NDP* gene, which encodes norrin, cause the rare X-linked disorder Norrie disease (OMIM #310600). Clinical manifestations of Norrie disease include retinal hypovascularization, retinal detachment, vitreous hemorrhages, and cataract, ultimately leading to blindness.^1^^,^^10^ Previous studies have shown that ectopic expression of high levels of norrin restores normal retinal capillary formation in *Ndp*-deficient mutant mice.^8^ Furthermore, ectopic overexpression of norrin in the lens or the retinal pigment epithelium (RPE) promotes vascular regrowth in *Ndp-*deficient mice while suppressing pathological neovascularization in a mouse model of oxygen-induced retinopathy (OIR).^11^ These findings indicate that norrin has strong potential not only to promote developmental intraretinal capillary growth but also to enhance vascular stability following premature oxygen exposure. Abnormal vessel growth is a hallmark of several retinal vascular diseases, including retinopathy of prematurity (ROP), diabetic retinopathy, and neovascular age-related macular degeneration (AMD). Physiological and pathological retinal angiogenesis is driven, among other factors, by vascular endothelial growth factor-A (VEGF-A). Therefore, the reduction of VEGF-A is a critical mechanism in therapies targeting retinal and choroidal neovascularization. Clinical studies demonstrate a robust beneficial effect of anti-VEGF-A therapy in preserving vision in neovascular AMD,^12^ whereas its benefit in ROP remains less conclusive.^13^^,^^14^ Complete inactivation of VEGF-A appears disadvantageous, as data from mutant mice indicate a critical role for endogenous VEGF-A, continuously released by the RPE, in the survival and maintenance of photoreceptors, Müller glia, and the choriocapillaris, the vascular bed essential for nourishing photoreceptors.^15^^,^^16^^,^^17^ Given these limitations, norrin offers a pro-angiogenetic alternative that may serve as a potential treatment for retinal vascular diseases.^18^^,^^19^^,^^20^

In recent years, AAV-mediated gene transfer has been emerged as a powerful approach to treat retinal disorders.^21^^,^^22^ Different adeno-associated viral (AAV) serotypes allow for cell type-specific expression, and the technology enables long-term expression of the target gene, reducing the need for repeated therapeutic injections. Here, we demonstrate that AAV-mediated norrin expression in the retina fully rescues the *Ndp*-deficient retinal phenotype in mice. This treatment promotes intraretinal capillary development, restores blood-retinal barrier integrity, and increases visual function. Moreover, AAV-mediated norrin expression stabilizes retinal vasculature and suppresses pathological neovascularization by reducing *Vegfa164* and *Ang-2* expression following oxygen treatment in mice.

## Results

### AAV2-7m8 and AAV-ShH10 serotypes transduce Müller glia and retinal ganglion cells in juvenile wild-type mice

**Figure 1:**
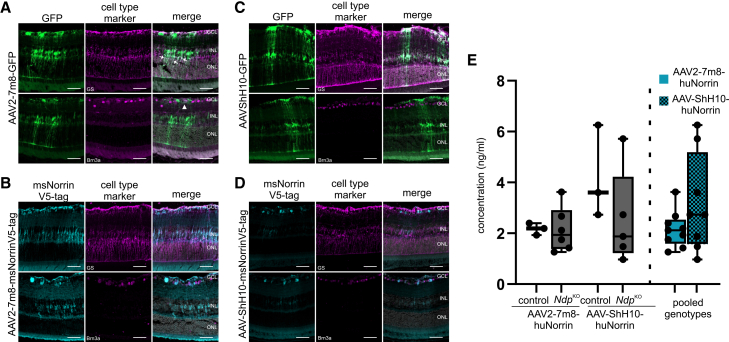
Tropism and transduction of AAV2-7m8 and AAV-ShH10 in the juvenile wild-type mouse retina (A) GFP expression (green) co-labeled with GS (magenta, upper) and Brn3a (magenta, lower) following intravitreal injection of AAV2-7m8-GFP. Nuclei stained with DAPI (gray). Scale bars: 50 μm. (B) V5-tag immunostaining (cyan) co-labeled with GS (magenta, upper) and Brn3a (magenta, lower) after intravitreal injection of AAV2-7m8-msNorrin-V5-tag. Nuclei, DAPI (gray). Scale bars: 50 μm. (C) GFP expression (green) co-labeled with GS (magenta, upper) and Brn3a (magenta, lower) after intravitreal injection of AAV-ShH10-GFP. Nuclei, DAPI (gray). Scale bars: 50 μm. (D) V5-tag immunostaining (cyan) co-labeled with GS (magenta, upper) and Brn3a (magenta, lower) after intravitreal injection of AAV-ShH10-msNorrin-V5-tag. Nuclei, DAPI (gray). Scale bars: 50 μm, *n* ≥ 3. (E) ELISA quantification of norrin protein in the neuronal retina after intravitreal injection of AAV2-7m8-huNorrin or AAV-ShH10-huNorrin in control and *Ndp*^KO^ mice. *n* = 3–5 (AAV2-7m8: control: 2.17 (0.23) ng/ml, *Ndp*^KO^ : 2.14 (0.89) ng/ml; AAV-ShH10: control: 4.20 (81.84) ng/ml, *Ndp*^KO^: 2.56 (1.88) ng/ml; AAV2-7m8 poolled: 2.15 (0.71) ng/ml, AAV-ShH10 pooled: 3.17 (1.93) ng/ml). Data represent mean (SD). Student’s *t* test was used for statistical analysis.

The tropism and transduction efficiency of AAV2-7m8^23^ and AAV-ShH10^24^ were evaluated in C57BL/6J wild-type mice (Figure 1). Intravitreal injections of either GFP-expressing AAV (Figures 1A and 1C) or murine norrin fused to a V5-tag (Figures 1B and 1D) were performed on postnatal day (P) 7. To assess tropism, retinae were analyzed at P28 by double immunostaining to detect glutamine synthetase (GS) for Müller glia (Figure 1A, upper) and Brn3a for retinal ganglion cells (Figure 1A, lower). Following AAV2-7m8 injection, Müller glia were the predominantly transduced cell type (Figure 1A). With AAV2-7m8-msNorrin-V5-tag, V5-tag expression was detected in both Müller glia (Figure 1B, upper) and retinal ganglion cells (Figure 1B, lower), confirmed by co-labeling with GS and Brn3a. AAV-ShH10 produced a different pattern: GFP was detected in Müller cells (Figure 1C, upper) but absent in retinal ganglion cells (Figure 1C, lower). However, when AAV-ShH10-msNorrin-V5-tag was used, strong V5 immunoreactivity was observed in retinal ganglion cells (Figure 1D, lower) and only weak staining in Müller cells (Figure 1D, upper). ELISA was performed to quantify retinal levels of human norrin after intravitreal AAV injection (Figure 1E). Samples of the neuronal retina were collected at P28 from either control or *Ndp*^KO^ mice injected with AAV2-7m8 or AAV-ShH10, both expressing human norrin. In control mice injected with AAV2-7m8, the concentration measured 2.17 (0.23) ng/mL and 2.14 (0.89) ng/mL in *Ndp*^KO^ mice. In contrast, injection of AAV-ShH10 resulted in 4.20 (1.84) ng/mL in control mice and 2.56 (1.88) ng/mL in *Ndp*^KO^ mice. When samples from both genotypes were pooled, retinal concentrations averaged 2.15 (0.71) ng/mL for AAV2-7m8 and 3.17 (1.93) ng/mL for AAV-ShH10. No norrin was detected in contralateral eyes injected with the respective AAV-stuffer control vectors (data not shown).

### AAV-mediated norrin expression restores capillary growth in *Ndp*-deficient mice

**Figure 2:**
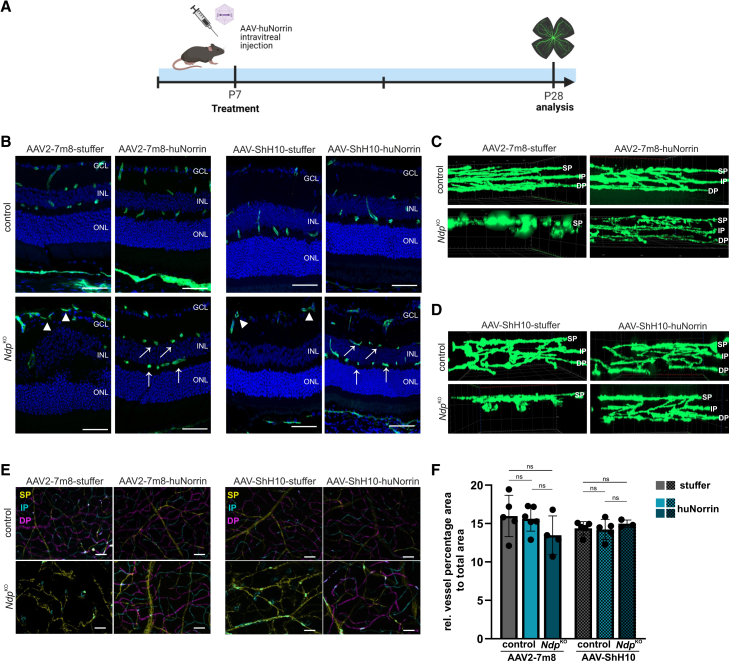
AAV-mediated norrin expression completely rescues intraretinal capillary formation in *Ndp*-deficient mice (A) Intravitreal injection of 3 × 10^9^ VG/μL of the AAV2-7m8 or AAV-ShH10 at P7 with analysis at P28. Contralateral eyes received the respective AAV-stuffer control. (B) Retinal capillaries visualized in FITC-dextran-perfused retinal cross sections (green). Nuclei, DAPI (blue). Scale bars: 50 μm. (C and D) Vertical view of 3D reconstructions from z stacks of FITC-dextran perfused retinal flat mounts. (E) Retinal flat mount showing all three retinal vascular plexuses. Superficial plexus (yellow), intermediate plexus (cyan), and deep plexus (magenta). Scale bars: 50 μm. (F) Quantification of vessel density in the deep vascular plexus expressed as the percentage of total area (*n* = 3–6) (control: AAV2-7m8-stuffer: 15.98 (2.69), control AAV-7m8-huNorrin: 15.62 (1.61); *Ndp*^KO^ AAV2-7m8-huNorrin: 13.47 ± 2.52; control: AAV-ShH10-stuffer: 14.37 (0.87), control AAV-shH10-huNorrin: 14.22 (1.31); *Ndp*^KO^ AAV-ShH10-huNorrin: 14.97 (0.48)). Data represented mean (SD). One-way ANOVA was used for statistical analysis.

To test whether AAV-mediated expression of human norrin could rescue the phenotype of *Ndp*-deficient mice, intravitreal injections were performed in *Ndp*-deficient mice and their control littermates (Figure 2A). Specifically, *Ndp*^KO^ mice received intravitreal injections of either AAV2-7m8-huNorrin or AAV-ShH10-huNorrin at P7, while the contralateral eye was injected with the corresponding stuffer AAV serotype. Control mice were injected in parallel with the same AAV serotypes. To visualize and analyze intraretinal capillaries, mice were perfused with high-molecular-weight FITC-conjugated dextran at P28, followed by preparations of meridional cross sections and retinal flat mounts. Analysis of retinal cross sections showed a normal distribution of the retinal plexuses in control mice, irrespective of whether they had received huNorrin-expressing or stuffer AAV injections (Figure 2B). In contrast, *Ndp*^KO^ mice injected with the stuffer AAV serotype exhibited the previously described phenotype: retinal vessels remained restricted to the superficial plexus (Figure 2B, arrowhead), displaying glomeruloid-like malformations and a complete absence of capillaries of both the deep and intermediate plexuses.^8^ Strikingly, in *Ndp*^KO^ mice injected with AAV2-7m8 or AAV-ShH10 expressing huNorrin, intraretinal capillaries of both the intermediate and deep vascular plexuses were clearly detectable in meridional sections (Figure 2B, arrow). These findings were confirmed by flat mount analysis (Figures 2C–2E). Three-dimensional reconstruction of z stacks through the neuronal retina revealed all three vascular plexuses with multiple interconnected vessels in *Ndp*^KO^ mice expressing huNorrin as well as in the two control groups. By contrast, *Ndp*^KO^ mice injected with stuffer AAV2-7m8 or AAV-ShH10 showed no rescue of the phenotype (Figures 2C and 2D). To assess the densities of the capillary beds within each retinal vascular plexus, capillaries were traced on retinal whole mounts, color coded, and superimposed to generate merged pictures. Using this approach, no obvious differences in capillary density were observed between control mice and huNorrin-expressing *Ndp*^KO^ mice (Figure 2E). For quantitative analysis, the vessel area percentage of the deep vascular plexus was determined (Figure 2F). To this end, eight pictures, two from each retinal quadrant, were acquired from flat mounts and analyzed using the AngioTool software. Since *Ndp*^KO^ mice injected with the stuffer AAV did not develop a deep vascular plexus, this group was excluded from the analysis. In control mice, AAV2-7m8-stuffer and huNorrin injections resulted in relative vessel area percentages of 15.98 (2.69) and 15.62 (1.61) (Figure 2F). *Ndp*^KO^ mice injected with AAV2-7m8-huNorrin showed a relative vessel area percentage of 13.47 ± 2.52. No significant differences were observed between groups. Similarly, control mice injected with AAV-ShH10-stuffer or huNorrin showed relative vessel area percentages of 14.37 (0.87) and 14.22 (1.31), respectively. *Ndp*^KO^ mice injected with AAV-ShH10-huNorrin showed a relative vessel area percentage of 14.97 (0.48). Again, no significant differences were detected between groups (Figure 2F).

### AAV2-7m8-mediated norrin expression preserves the blood-retinal barrier in *Ndp*-deficient mice

**Figure 3:**
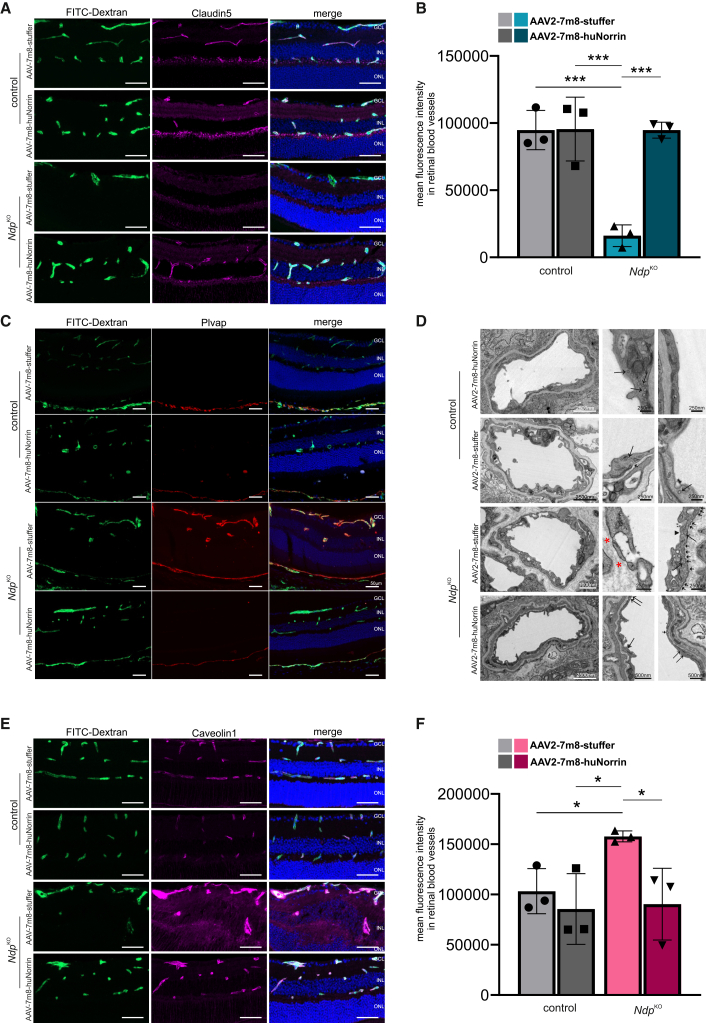
AAV-mediated norrin expression restores an intact blood-retinal barrier in *Ndp*-deficient mice (A) Immunohistochemical staining of claudin-5 (magenta) in FITC-dextran-perfused retinal gross sections (green) from control and *Ndp*^KO^ mice at P28 after intravitreal injection of AAV2-7m8-stuffer or AAV2-7m8-huNorrin at P7. Nuclei, DAPI (blue). Scale bars: 50 μm. (B) Quantification of claudin-5 mean fluorescence intensity in retinal blood vessels (*n* = 3) (beam intensity control-stuffer: 94851 (14616); control-huNorrin: 95505 (23752); *Ndp*^KO^-stuffer: 16126 (8110); *Ndp*^KO^-huNorrin 94756 (5965)). (C) Immunostaining of PLVAP (red) in FITC-dextran-perfused retinal cross sections (green) from control and *Ndp*^KO^ mice at P28 following AAV2-7m8-stuffer or AAV2-7m8-huNorrin injection at P7. Nuclei, DAPI (blue). (D) Transmission electron microscopy of retinal blood vessels from control and *Ndp*^KO^ mice at P28 following AAV2-7m8-stuffer or AAV2-7m8-huNorrin injection at P7. (E) Immunostaining of caveolin-1 (magenta) in FITC-dextran-perfused retinal cross sections from control and *Ndp*^KO^ mice at P28 after AAV2-7m8-stuffer or AAV2-7m8-huNorrin injection at P7. Nuclei, DAPI (blue). Scale bars: 50 μm. The image control AAV2-7m8-huNorrin was reused from Figure 2B. (F) Quantification of caveolin-1 mean fluorescence intensity in retinal blood vessels (*n* = 3). Scale bars: 50 μm (beam intensity control-stuffer: 103327 (22389); control-huNorrin: 85566 (35129); *Ndp*^KO^-stuffer: 157801 (5425); *Ndp*^KO^-huNorrin 90362 (35641)). All data are mean (SD). One-way ANOVA was used for statistical analysis. ∗*p* ≤ 0.05, ∗∗∗*p* < 0.001.

To determine whether huNorrrin-induced capillaries exhibit the biological properties of wild-type retinal capillaries, immunohistochemistry was performed using antibodies against claudin-5, plasmalemma-vesicle-associated protein (PLVAP), and caveolin-1 (Figure 3). Claudin-5 serves as a marker for tight junctions, which are essential for maintaining the blood-retinal barrier. PLVAP forms diaphragms in fenestrated capillaries such as those of the choriocapillaris and is expressed by the leaky fenestrated superficial capillaries of *Ndp*^KO^ mice. Caveolin-1 is highly expressed in fenestrated capillaries but only weakly in retinal vessels with an intact blood-brain barrier. Immunohistochemical analysis of claudin-5 revealed an intact blood-retinal barrier in AAV2-7m8-huNorrin-injected *Ndp*^KO^ mice (Figure 3A) as well as in control mice injected with AAV2-7m8-stuffer or AAV2-7m8-huNorrin. In contrast, claudin-5 expression was completely absent in *Ndp*^KO^ mice injected with the stuffer AAV. The findings were corroborated by quantification of claudin-5 immunoreactivity (beam intensity control-stuffer: 94,851 [14,616]; control-huNorrin: 95,505 [23,752]; *Ndp*^KO^-stuffer: 16,126 [8,110]; *Ndp*^KO^-huNorrin: 94,756 [5,965]) (Figure 3B). The presence of fenestrae in stuffer-injected *Ndp*^KO^ mice was confirmed by immunohistochemical staining for PLVAP (Figure 3C). In control mice injected with either AAV2-7m8-stuffer or AAV2-7m8-huNorrin, PLVAP expression was restricted to the choriocapillaris (Figure 3C). Similarly, no PLVAP was detected in the retinal capillaries of AAV2-7m8-huNorrin-injected *Ndp*^KO^ mice (Figure 3C). In control mice injected with AAV2-7m8-stuffer or AAV2-7m8-huNorrin, caveolin-1 was detected in FITC-labeled retinal capillaries. By contrast, *Ndp*^KO^ mice injected with AAV2-7m8-stuffer showed a dramatic increase in caveolin-1 signal, indicating an enhanced transcytosis in the leaky, fenestrated superficial capillaries. Intriguingly, in AAV2-7m8-huNorrin-injected *Ndp*^KO^ mice, caveolin-1 immunoreactivity was reduced to levels comparable to those in control groups (Figure 3E). This was confirmed by quantification of caveolin-1 immunoreactivity (beam intensity control-stuffer: 103,327 [22,389]; control-huNorrin: 85,566 [35,129]; *Ndp*^KO^-stuffer: 157,801 [5,425]; *Ndp*^KO^-huNorrin: 90,362 [35,641]) (Figure 3F). Transmission electron microscopy (TEM) was performed to examine the ultrastructure of the retinal capillaries (Figure 3D). In stuffer-injected *Ndp*^KO^ mice, fenestrae (Figure 3D, asterisk) and numerous transcytotic vesicles (Figure 3D, arrow and arrowhead) were observed. In contrast, AAV2-7m8-huNorrin-injected *Ndp*^KO^ mice showed a complete absence of fenestrae, with only a few transcytotic vesicles present (Figure 3D). Additionally, AAV-mediated norrin expression reduced the reactivity of astrocytes, Müller cells, and microglia (Figure S2).

### AAV-mediated norrin expression preserves visual function in *Ndp*-deficient mice

**Figure 4:**
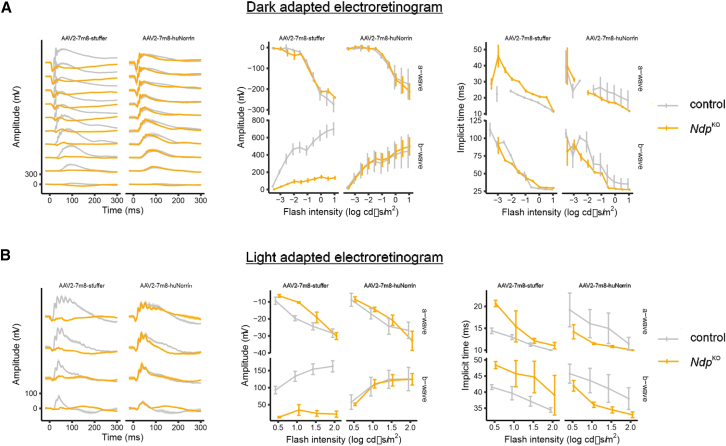
AAV-mediated norrin expression restores visual function in *Ndp*-deficient mice (A and B) Dark-adapted rod (A) and light-adapted cone ERG (B) waveforms and peak amplitudes of *in vivo* electroretinogram recordings of control and *Ndp*^KO^ mice at P28 after intravitreal injection of AAV2-7m8-stuffer or AAV2-7m8-huNorrin at P7.

To analyze whether huNorrrin-induced capillary formation leads to restoration of the visual function in *Ndp*-deficient mice, we performed functional electroretinogram (ERG) analyses. In Figure 4, ERG waveforms (dark-adapted A; light-adapted B) are shown on the left, alongside their averages with standard deviation in responses to single flashes of increasing intensity. In both dark-adapted (A) and light-adapted (B) ERGs, AAV2-7m8-stuffer *Ndp*-deficient mice exhibited waveforms in which b-wave amplitudes were smaller than the a-wave amplitudes, indicative of a negative ERG waveform. This pattern reflects a selective reduction or dysfunction of bipolar cells and their downstream signaling, while photoreceptors remain largely intact. Negative ERGs have also been observed in ROP, where abnormal retinal vasculature supplies the inner retina, but photoreceptors are nourished by oxygen from the choroid.^25^ In contrast, AAV2-7m8-huNorrin-injected *Ndp*-deficient mice showed comparable a- and b-wave amplitudes to both AAV2-7m8-stuffer-injected and AAV2-7m8-huNorrin-injected control mice. Furthermore, AAV-ShH10-mediated norrin expression significantly restored visual function in *Ndp*-deficient mice (Figure S3).

### scAAV2-7m8 tropism and transduction in the juvenile retina

**Figure 5:**
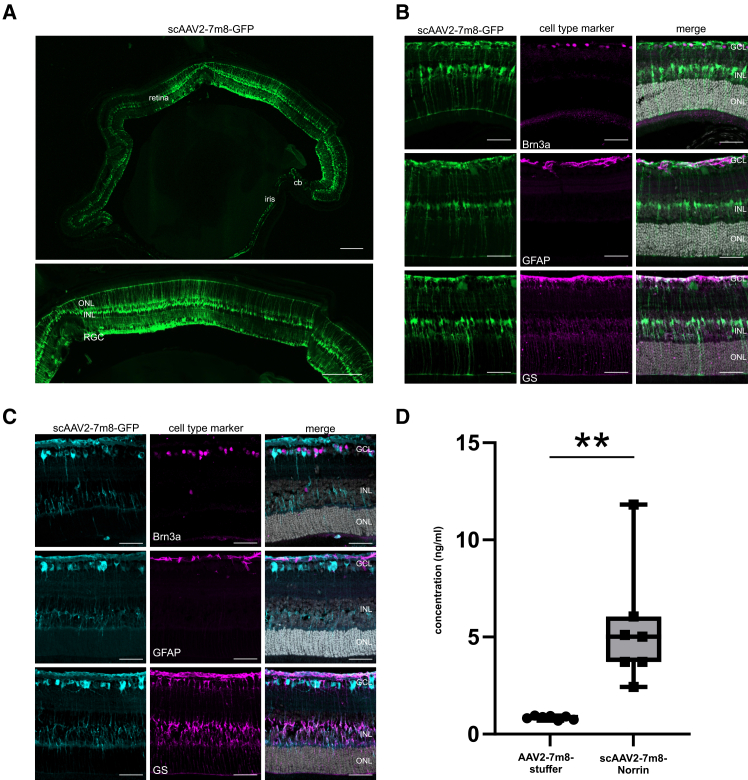
Tropism and transduction of dsAVV2-7m8 in the juvenile mouse retina (A) GFP expression (green) in the whole retina at P17 after dsAAV2-7m8-GFP intravitreal injection at P7. Scale bars: 200 μm. (B and C) GFP expression (green) in retinal cross sections double stained with Brn3a (magenta, upper), GFAP (magenta, middle), and GS (magenta, lower) at P17 following dsAAV2-7m8-GFP injection at P7. Scale bars: 50 μm. (C) Immunohistochemical staining against V5-tag (cyan) double stained with Brn3a (magenta, upper), GFAP (magenta, middle), and GS (magenta, lower) at P17. Scale bars: 50 μm. (D) ELISA measurement of the norrin concentration in the sensory retina at P17 after dsAAV2-7m8-huNorrin or AAV2-7m8-stuffer injection at P7. *n* = 6 (scAAV-7m8-stuffer: 0.84 (0.10) ng/ml; scAAV-7m8-huNorrin: 5.41 (3.06) ng/ml). All data represented as mean (SD). Student’s *t* test was used for statistical analysis. ∗∗*p* < 0.01.

For the following experiments in the OIR model, we used AAV2-7m8 vectors harboring a self-complementary (sc) expression cassette. In contrast to the first part of this study, the time from AAV injection to final analysis in the OIR model was only 10 days. Therefore, we validated the tropism and transduction efficiency of scAAV2-7m8 in this context (Figure 5). GFP-expressing scAAV2-7m8 was intravitreally injected into wild-type mice at P7, and retinae were analyzed on P17. Cross-sectional analysis revealed nearly complete transduction throughout the retina (Figure 5A). To determine the transduced cell types, we performed immunohistochemical co-staining with cell-specific markers. Only minimal colocalization of GFP with Brn3a-positive retinal ganglion cells was observed (Figure 5B, upper). In contrast, strong GFP expression was detected in retinal astrocytes and Müller cells with GFAP (Figure 5B, middle) and GS (Figure 5B, lower), respectively, confirming efficient transduction of retinal glia cells. To assess the localization of norrin after the AAV-mediated expression, we injected AAV2-7m8 encoding msNorrin fused to a V5-tag (Figure 5C). Immunohistochemical staining for V5 and Brn3a clearly showed that retinal ganglion cells expressed norrin (Figure 5C, upper). Furthermore, we showed that astrocytes, stained for GFAP (Figure 5C, middle), and Müller cells, stained for GS (Figure 5C, lower), expressed norrin following intravitreal AAV injection. Finally, we quantified retinal norrin levels by ELISA following scAAV2-7m8-huNorrin injection. Norrin concentrations reached 5.41 (3.06) ng/mL compared with 0.84 (0.10) ng/mL in stuffer-injected controls (Figure 5D).

### Norrin expression via AAV2-7m8 enhances vascular stability and reduces neovascularization in OIR

**Figure 6:**
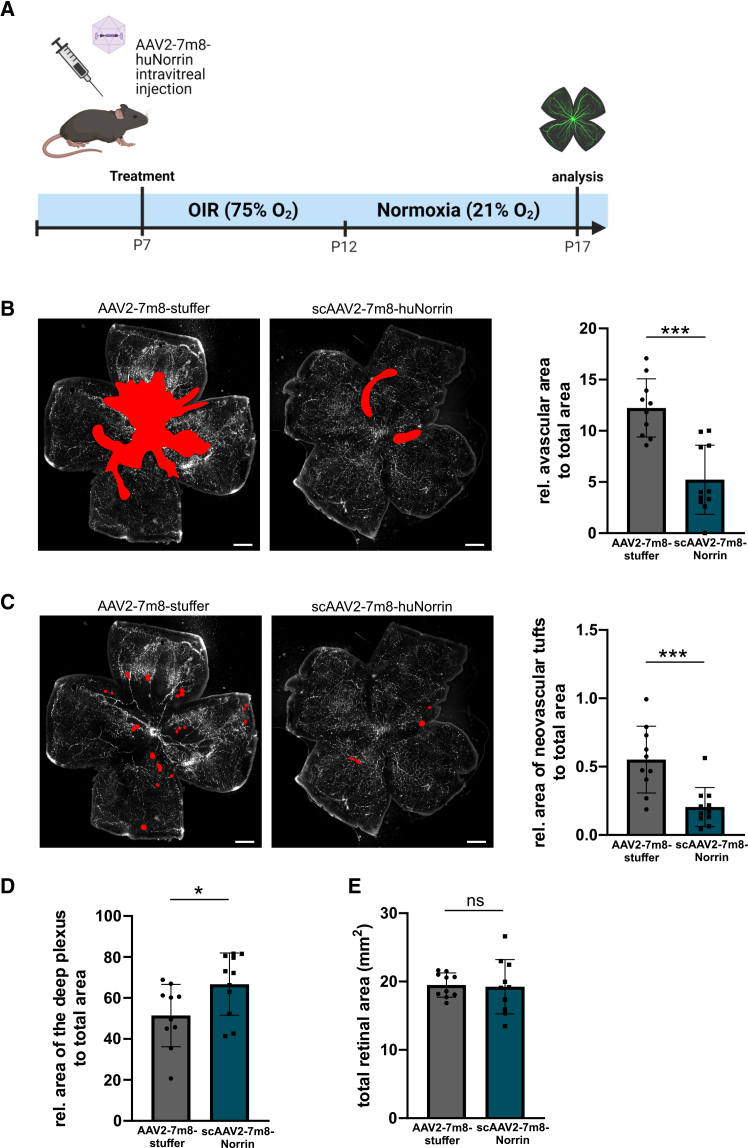
AAV-mediated expression of norrin promotes vascular stability, by increased vascular regrowth and suppressed formation of neovascular tufts (A) 3 × 10^9^ VG/μL of the dsAAV2-7m8 was injected intravitreally on P7. The contralateral eye served as a control and was injected with the respective AAV-stuffer serotype. Experimental mice were kept under 75% O_2_ for 5 days and returned to room air (21% O_2_) until final analysis on P17. (B) Exemplary images of FITC-dextran-perfused retinal flat mounts. Red area indicates the avascular area. Quantification of relative avascular area percentage blotted to total retinal area (stuffer: 12.23 (2.85) %, huNorrin: 5.22 (3.38) %). (C) Exemplary images of FITC-dextran-perfused retinal flat mounts. Red areas indicate the area of neovascular tufts. Quantification of relative percentage area of neovascular tufts blotted to total retinal area (stuffer: 0.55 (0.24) %, huNorrin: 0.20 (0.14) %). (D) Quantification of relative area of the deep vascular plexus to total retinal area (stuffer: 51.42 (15.19) %; huNorrin: 66.74 (15.21) %). (E) Total retinal flat mount area. Scale bars: 500 μm. *n* = 9–11. All data represented as mean (SD). Student’s *t* test was used for statistical analysis. ∗*p* ≤ 0.05, ∗∗∗*p* < 0.001.

To assess whether AAV-mediated norrin expression promotes vascular stability and modulates neovascular tuft formation after oxygen exposure, we performed the OIR model in wild-type mice injected intravitreally with AAV2-7m8-stuffer or scAAV2-7m8-huNorrin at P7, followed by exposure to 75% O_2_ for 5 days and return to room air until P17 (Figure 6A). Retinal flat mounts perfused with FITC-dextran were analyzed to determine the vaso-obliterated area. AAV2-7m8-huNorrin significantly reduced the vaso-obliterated area to 5.22% (3.38%) compared with 12.23% (2.85%) in stuffer controls (Figure 6B). Neovascular tuft formation in the superficial plexus was also reduced by norrin expression (0.20% [0.14%]) relative to controls (0.55% [0.24%]; Figure 6C). In addition, the area of the deep vascular plexus was significantly increased in huNorrin-treated mice (66.74% [15.21%]) compared with controls (51.42% [15.19%]) (Figure 6D). Total retinal area did not differ between groups (stuffer: 19.48 [1.77] mm^2^; huNorrin: 19.24 [3.98] mm^2^; Figure 6E).

### AAV2-7m8-mediated norrin expression reduced Vegfa164 and Ang-2 expression following oxygen treatment

**Figure 7:**
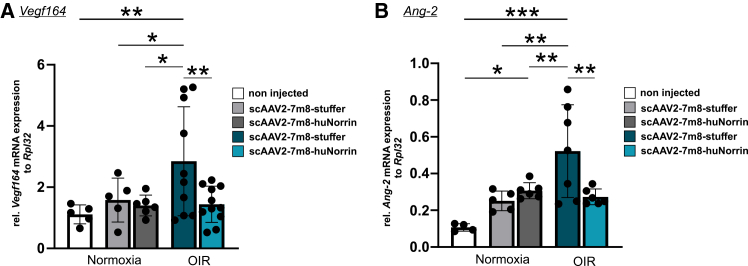
AAV-mediated expression of norrin reduced Vegfa164 and Ang-2 expression after oxygen treatment (A) Real-time RT-PCR analysis of *Vegfa*164 in the sensory retina at P15 after intravitreal AAV injection at P7 and oxygen treatment (P7–P12). *Rpl*32 was used as a housekeeping gene. All data represented as mean ± SD. One-way ANOVA was used for statistical analysis. ∗*p* ≤ 0.05, ∗∗*p* < 0.01 (Normoxia: uninjected: 1.11 (0.31); dsAAV2-7m8-stuffer: 1.58 (0.72); scAAV2-7m8-huNorrin: 1.40 (0.34); OIR: AAV2-7m8-stuffer – OIR: 2.85 (1.78); scAAV2-7m8-huNorrin – OIR: 1.44 (0.59)). (B) Real-time RT-PCR analysis of *Ang*-2 in the sensory retina at P15 after intravitreal AAV injection at P7 and oxygen treatment (P7–P12). *Rpl*32 was used as a housekeeping gene. All data represented as mean (SD). One-way ANOVA was used for statistical analysis (Normoxia: non-injected: 0.11 (0.02); scAAV2-7m8-stuffer: 0.25 (0.05); scAAV2-7m8-huNorrin: 0.31 (0.04); OIR: AAV2-7m8-stuffer - OIR: 0.52 (0.25); scAAV2-7m8-huNorrin – OIR: 0.27 (0.04)). ∗*p* ≤ 0.05, ∗∗*p* < 0.01, ∗∗∗*p* < 0.001.

To investigate the molecular mechanism underlying the norrin-mediated effects on vascular stability after oxygen treatment, we analyzed mRNA expression of *Vegfa164* and *angiopoietin*-2 (Ang-2) in the neuronal retina at P15 following AAV injection and OIR (Figure 7). Control groups included non-injected normoxic mice and AAV-injected mice maintained under normoxia. *Vegfa164* mRNA expression was unchanged under normal conditions across all groups (uninjected: 1.11 [0.31]; dsAAV2-7m8-stuffer: 1.58 [0.72]; scAAV2-7m8-huNorrin: 1.40 [0.34]; Figure 7A). Following oxygen treatment, stuffer-injected mice showed a significant increase in *Vegfa164* mRNA expression compared with all normoxic control groups (AAV2-7m8-stuffer, OIR: 2.85 [1.78]; Figure 7A). In contrast, AAV-mediated norrin expression significantly reduced the *Vegfa164* mRNA levels after OIR to values comparable to control groups (scAAV2-7m8-huNorrin, OIR: 1.44 [0.59]; Figure 7A). Analysis of *Ang*-2 mRNA expression revealed that dsAAV2-7m8-stuffer injection under normoxic conditions did not alter Ang-2 levels compared with non-injected animals (non-injected: 0.11 [0.02]; scAAV2-7m8-stuffer: 0.25 [0.05]; Figure 7B). Interestingly, AAV-mediated expression of huNorrin led to a significant increase in *Ang*-2 mRNA levels under normoxic oxygen conditions compared with non-injected or stuffer-injected animals (scAAV2-7m8-huNorrin: 0.31 [0.04]; Figure 7B). Similar to *Vegfa*164, *Ang*-2 mRNA expression was significantly elevated in dsAAV2-7m8-stuffer-injected mice after OIR, compared with all three control groups (AAV2-7m8-stuffer, OIR: 0.52 [0.25]; Figure 7B). In contrast, AAV-mediated huNorrin expression following oxygen treatment significantly reduced *Ang*-2 mRNA expression relative to the stuffer-injected OIR group (scAAV2-7m8-huNorrin, OIR: 0.27 [0.04]; Figure 7B).

## Discussion

We conclude that AAV-mediated retinal norrin expression fully rescues the *Ndp*-deficient retinal phenotype, restores vascular integrity, and suppresses pathological neovascularization following OIR in mice, highlighting the translational potential of norrin gene therapy for retinal vascular disease. This conclusion is supported by (1) restoration of intraretinal capillary development in both the inner and outer plexiform layers of *Ndp*-deficient mice, (2) recovery of blood-retinal barrier integrity and normal ERG function, (3) reduced avascular zones and neovascular areas together with increased deep vascular plexus regeneration after oxygen injury, and (4) decreased expression of *Vegfa164* and *Ang-2* after oxygen treatment, suggesting coordinated modulation of angiogenic signaling and vascular repair.

Abnormal angiogenesis and vascular leakage are hallmarks of many retinal vascular diseases. Elevated VEGF-A levels drive excessive blood vessel formation and, through disruption of the blood-retinal barrier, contribute to increased vascular permeability and retinal edema. Current treatments for diseases such as ROP, diabetic retinopathy, macular edema, and AMD rely on anti-VEGF-A therapies and represent the current standard of care. However, these approaches have important limitations including the need for repeated intravitreal injections, treatment burden, dependence on patient adherence and retreatment timing, and variable therapeutic responses. In addition, sustained VEGF suppression may interfere with physiological vascular and neuronal homeostasis.

Using AAV-mediated norrin expression, we achieved complete restoration of retinal vascularization, preservation of blood-retinal barrier integrity, and maintenance of visual function in *Ndp*-deficient mice. Whereas formation of the superficial retinal vascular plexus is driven primarily by VEGF-A gradients at the retinal surface, sprouting of endothelial cells into the retina to generate the deep and intermediate plexuses depends predominantly on Norrin/FZ4/β-catenin signaling.^4^^,^^5^^,^^6^^,^^7^^,^^9^^,^^26^^,^^27^^,^^28^^,^^29^^,^^30^^,^^31^^,^^32^ As norrin is predominantly expressed by Müller cells,^33^ restoration of norrin expression in this biologically relevant cell type appears critical for therapeutic efficacy. Consistent with this concept, both AAV serotypes used in this study predominantly transduced Müller cells, thereby re-establishing the endogenous signaling axis. Importance of cell specificity is further supported by our previous findings that ectopic norrin overexpression in the retinal pigmented epithelium failed to rescue the norrin-deficient phenotype,^34^ despite improving vascular regeneration following OIR.^8^

At the cellular level, norrin promotes proliferation, survival, migration, and tube formation through Lrp5/6-dependent signaling.^11^ In addition, ANG-2 appears to act as an important downstream mediator of retinal angiogenesis. *Ang-2* expression peaks during physiological sprouting of retinal vessels, and its deficiency prevents the formation of the deep and intermediate vascular plexuses and abolishes oxygen-induced neovascularization.^35^^,^^36^ Together with previous observations that norrin induces *Ang-2* expression in retinal endothelial cells,^11^ our findings support a model in which coordinated norrin/ANG-2 signaling contributes to physiological vascular regeneration. Importantly, despite its potent pro-angiogenic activity, additional AAV-mediated norrin expression in wild-type mice did not induce aberrant vessel growth, suggesting that norrin restores normal vascularization rather than uncontrolled pathological neovascularization.

In the mouse OIR model, the hyperoxic vaso-obliterative phase occurs between P7 and P12, followed by a hypoxia-driven neovascular and regenerative phase from P12 to P17. Because our experimental design required intravitreal injection at P7 with analysis at P17, we employed a self-complementary AAV vector to maximize the onset of vector-derived norrin expression. Nevertheless, retinal transgene expression from self-complementary vectors generally becomes detectable only several days after injection,^37^ with broader protein expression typically occurring approximately 5–10 days after delivery, corresponding to around P12–P14 in our study. Therefore, it appears unlikely that vector-derived norrin reached levels sufficient to substantially prevent the initial hyperoxia-induced vaso-obliteration. This interpretation is supported by the concurrent reduction in VEGF-A and ANG-2 expression in AAV-treated mice, consistent with improved vascular regeneration and reduced hypoxic stress. However, we cannot exclude that low early levels of vector-derived norrin contributed partially to protection against vascular loss during the vaso-obliterative phase.

While the present study demonstrates robust therapeutic efficacy following treatment at P7, this intervention precedes the time point at which treatment would typically be initiated in patients with ROP. We selected this early injection paradigm to ensure sufficient AAV-mediated transgene expression during the neovascular phase of OIR, given the delayed onset of retinal transgene expression after AAV delivery. Notably, the pronounced regenerative effects observed despite these temporal constraints highlight the translational potential of this approach.

Our findings therefore represent an important step toward the development of AAV-mediated norrin therapy for retinal vascular disease. Sustained retinal norrin expression may provide therapeutic advantages; however, comprehensive long-term evaluation will be essential to establish safety and clinical relevance. Future studies should investigate the effects of prolonged norrin expression on retinal structure, vascular integrity, inflammatory responses, and visual function. Encouragingly, published data from genetic models of retinal norrin overexpression support the feasibility and therapeutic potential of sustained activation of norrin signaling in the retina.^8^

In retinal vascular disease, tissue damage is driven not only by abnormal vessel growth but also by the formation of non-functional, leaky vessels, making restoration of vascular barrier integrity an important therapeutic objective. In *Ndp*-deficient mice, vascular dysfunction characterized by reduced claudin-5 expression, increased transcytosis, and endothelial fenestrations was fully rescued by AAV-mediated norrin expression. Because retinal vascular architecture was simultaneously restored, the improvement in blood-retinal barrier function may, at least in part, reflect normalization of VEGF-A levels. In *Ndp-*deficient mice, hypoxia-induced VEGF-A upregulation likely acts as a compensatory response to absent retinal vasculature but also contributes to barrier disruption. Consistent with this interpretation, norrin has previously been shown to restore blood-retinal barrier integrity even after VEGF-induced vascular leakage,^38^ and the Wnt target gene *MFSD2A* has been identified as a partial regulator of vascular transcytosis in *Ndp*- and *Lrp5*-deficient mice.^39^

Notably, additional AAV-mediated norrin expression in wild-type mice did not alter claudin-5 and caveolin-1 signal intensity. This further suggests that elevated norrin levels in a healthy retina do not disrupt vascular endothelial development and structure. It is of interest to note that, despite increased PLVAP expression and the presence of endothelial fenestrae in *Ndp*-deficient mice, we did not observe overt FITC-dextran leakage under the experimental conditions used. This may, at least in part, be explained by the relatively high molecular weight and hydrodynamic size of the FITC-dextran tracer employed in this study. Although endothelial fenestrae exhibit an ultrastructural diameter of approximately 60–80 nm, the presence of diaphragms and glycocalyx-associated filtration barriers generally restricts efficient passage to substantially smaller solutes.^40^ Consequently, permeability changes sufficient to permit diffusion of small molecules may still be insufficient to allow detectable extravasation of high-molecular-weight FITC-dextran.

The incidence of ROP is increasing as advances in neonatal care improve the survival of preterm infants.^41^ In these infants, retinal vascularization remains incomplete, and disease severity correlates closely with the degree of prematurity. ROP is initiated when preterm infants are exposed to relatively high oxygen levels after birth, resulting in retinal hyperoxia and vaso-obliteration of immature retinal capillaries. This first phase is characterized primarily by suppression of VEGF-A expression. During the second phase, the metabolically active developing retina becomes relatively hypoxic, triggering HIF-1α-dependent upregulation of VEGF-A together with additional pro-angiogenic mediators. These factors drive pathological neovascularization into the vitreous, ultimately leading to retinal traction and detachment. ANG-2, which is induced by both hypoxia and VEGF-A signaling, is also elevated in the vitreous of patients with advanced ROP.^42^^,^^43^ Consistently, combined inhibition of Tie-2, the receptor for ANG-2, and VEGF-A effectively suppresses pathological neovascularization in the OIR mouse model.^44^ In the present study, AAV-mediated norrin expression reduced *Vegfa164* and *Ang-2* mRNA levels following oxygen-induced stress and was associated with a marked decrease in pathological neovascularization. These findings indicate that coordinated regulation of *Vegfa164* and *Ang-2* contributes substantially to the anti-angiogenic effects of norrin in the OIR model. Given the established roles of VEGF-A and ANG-2 in hypoxia-induced vascular destabilization and sprouting angiogenesis, concomitant reduction of both factors is consistent with enhanced vascular normalization and alleviation of ischemic stress.

Notably, under normoxic conditions, AAV-mediated norrin delivery increased *Ang-2* expression. This observation suggests that ANG-2 may not solely represent a marker of vascular destabilization in this setting. Although ANG-2 is classically linked to vessel regression and pathological angiogenesis, emerging evidence indicates that its function is highly context dependent and may additionally involve vascular remodeling, endothelial plasticity, and adaptive reorganization of the vascular network.^45^ In the context of enhanced norrin signaling, increased Ang-2 expression may therefore reflect active remodeling or maturation of the retinal vasculature rather than destabilization alone. This interpretation supports the concept that norrin signaling influences endothelial biology beyond simple vascular stabilization, potentially promoting regenerative remodeling programs required for restoration of the retinal vascular network. Accordingly, the beneficial effects of norrin in the OIR model likely arise not only from suppression of pathological angiogenic signaling but also from the promotion of coordinated vascular repair and remodeling processes.

In contrast to current anti-VEGF therapies, norrin treatment restores *Vegfa164* expression toward physiological levels rather than inducing broad suppression. This distinction may be particularly relevant in preterm infants, in whom VEGF-A is required for normal retinal vascular development, neuronal survival, and tissue homeostasis. Indeed, retinal VEGF-A blockade has been associated with increased apoptosis of retinal pigmented epithelial cells, Müller glia, and photoreceptors.^46^^,^^47^^,^^48^ Beyond the retina, VEGF also exerts important neuroprotective effects in the central nervous system, including protection of hippocampal neurons against glutamate-induced excitotoxicity and hypoxic injury.^49^^,^^50^ Thus, therapeutic strategies that normalize rather than abolish VEGF-A signaling may provide a more favorable balance between suppression of pathological angiogenesis and preservation of physiological developmental processes.

An additional advantage of AAV-mediated norrin delivery is the potential for sustained long-term expression, particularly in postmitotic retinal cells, thereby reducing the need for repeated intravitreal injections. This feature could be especially advantageous in vulnerable preterm infants, for whom repeated ocular interventions carry procedural and anesthetic risks. Nevertheless, comprehensive long-term evaluation will be critical for the translational development of this therapeutic strategy. Future studies should therefore assess the long-term impact of AAV-mediated norrin therapy on retinal architecture, vascular integrity, inflammatory responses, neuronal survival, and visual function.

In a previous pilot study, we demonstrated the therapeutic potential of intravitreal recombinant norrin administration in the mouse OIR model.^11^ More recently, Chidiac and colleagues reported comparable findings using a Norrin/Wnt surrogate antibody designed to activate the FZD4:LRP5 receptor complex and thereby mimic norrin signaling.^18^ Although this strategy improved vascular abnormalities and restored blood-retina barrier integrity in OIR, surrogate receptor activation may not fully reproduce the spatial and temporal signaling dynamics of endogenous norrin. In addition, maintenance of therapeutic efficacy requires repeated administrations. By contrast, AAV-mediated norrin delivery enables sustained local expression following a single intravitreal injection, potentially providing prolonged therapeutic benefit while reducing the treatment burden and risks associated with repeated intraocular procedures.

Additional proof of concept for norrin-based therapy was provided by Pauzuolyte et al.,^19^ who demonstrated that systemic AAV9-mediated norrin delivery restored developmental retinal vascularization and cochlear function in an *Ndp*-deficient mouse model of Norrie disease.^19^ While both studies highlight the therapeutic potential of norrin signaling, our work extends these findings to a distinct and clinically relevant context by targeting acquired ischemic retinal pathology in the OIR model. In contrast to systemic administration, intravitreal delivery using the AAV2-7m8 capsid enables localized retinal gene transfer while minimizing systemic vector exposure, thereby potentially reducing off-target effects and systemic immune activation. Importantly, intravitreal AAV2-7m8 delivery efficiently targets Müller glia, the endogenous source of norrin in the retina, thereby restoring norrin signaling within its physiological cellular niche while avoiding widespread systemic exposure. Moreover, whereas the study by Pauzuolyte et al. primarily focused on structural and functional rescue, our work provides additional mechanistic insight through the analysis of *Vegfa164* and *Ang-2* regulation, together with assessments of vascular permeability and endothelial ultrastructure. Collectively, these findings support local AAV-mediated norrin delivery as a promising strategy for sustained retinal vascular repair with favorable translational and safety profiles.

Collectively, these findings identify targeted AAV-mediated ocular norrin delivery as a promising therapeutic approach for retinal vascular disease by simultaneously promoting vascular repair, maintaining vascular integrity, and preserving retinal function. Unlike current anti-VEGF approaches that primarily suppress angiogenic signaling, norrin appears to support physiological vascular repair through coordinated modulation of endothelial signaling pathways, including normalization of *Vegfa164* and *Ang-2* expression and restoration of blood-retinal barrier function. Importantly, local intravitreal delivery using an AAV2-7m8 vector enables sustained retinal expression while minimizing systemic exposure and reducing the need for repeated interventions. Together with previous studies targeting Norrin signaling, our results extend the therapeutic concept from congenital vascular defects to acquired ischemic retinal pathology and provide mechanistic insight into how Norrin promotes vascular recovery. Although further studies are required to establish long-term safety and define the therapeutic window in clinically relevant settings, our data support AAV-mediated norrin expression as a potentially translatable strategy for achieving durable and regenerative treatment of retinal vascular disease.

## Materials and methods

### Mice

Mice were housed under standardized conditions at 21°C room temperature (RT) and 62% relative air humidity. Feeding was *ad libitum*. Mice were kept at a 12 h light/dark cycle (6 a.m.–6 p.m.). All procedures conformed to the tenets of the National Institutes of Health Guidelines on the Care and Use of Animals in Research, the EU Directive 2010/63/E, and the ARVO Statement for the Use of Animals in Ophthalmic and Vision Research and were approved by the local authority (Regierung von Unterfranken, reference nos. 55.2-2532-2-793 and 55.2-2532-2-1463). *Ndp*^tm2Nat^ (The Jackson Laboratory, stock no. 012287)^7^ and C57BL/6 wild-type mice (Charles River) were used in this study. In all experiments, both sexes were included. *Ndp*^WT^ and *Ndp* ^+/−^ mice were used as control mice, as the phenotypical constitution of the vascular network in heterozygous mice does not differ to that of wild-type mice (see Figure S1). For simplicity, in the following sections, we will refer to *Ndp*^y/−^ and *Ndp*^−/−^ as *Ndp*^KO^ mice.

### Experimental procedures

Recombinant AAV2-7m8 or -ShH10 vectors containing either single-stranded or self-complementary expression constructs for norrin or GFP under the control of a CAG promoter, or non-containing stuffer DNA,^51^ were produced by transient transfection in HEK293 cells and purified as described previously.^52^ Intravitreal injections of different AAV serotypes were performed on P7 under isoflurane anesthesia. 1 μL (3.6 × 10^9^ VG/μL) of the respective serotype was injected. The contralateral eye served as a control and was injected with the respective control AAV. *Ndp*^KO^ and control mice were housed under room air until P28. For the OIR model, mice were placed after the intravitreal injection in a chamber containing 75% O_2_ for 5 days and afterward returned to room air until the final analysis on P15 or P17. For visualization of retinal blood vessels, mice were perfused through the left ventricle with 1 mL of PBS that contained 50 mg high molecular weight (MW = 2,000,000 g/mol) FITC-dextran (TdB Consultancy, Uppsala, Sweden). For retinal flat mounts, eyes were enucleated and placed in 4% paraformaldehyde (PFA) for 1 h. Retinae were dissected and flat mounted with Mowiol mounting medium. For immunohistochemistry, eyes were enucleated and either fixed with 4% PFA for 24 h or with 10% glacial acetic acid, 60% methanol, and 30% chloroform for 4 h at RT; transferred to 50% and 25% methanol 10 min each; and washed with 0.1 M phosphate buffer (Php). Eyes were equilibrated in 10%, 20%, and 30% sucrose, embedded in Tissue-Tek optimal cooling compound (Sakura Finetek Europe B.V., Zoeterwoude, Netherlands), and stored at −20°C.

### Immunofluorescence staining

Frozen sections were cut on a cryostat; incubated with 2% bovine serum albumin, 0.2% cold water fish gelatin, and 0.1% Triton X-100 in 0.1 M Php; and stained with the specific antibodies as follows: rat anti-PLVAP (1:50; Santa Cruz, Santa Cruz, USA), rabbit anti-caveolin1 (1:100; Cell Signaling Technology, Danvers, USA), rabbit anti-claudin5 (1:100; Invitrogen, Carlsbad, USA), guinea pig anti-Brn3a (1:1,000, Synaptic Systems), rabbit anti-V5-tag (1:100; Cell Signaling Technology), chicken anti-GFAP (1:1,000, LSBio), goat anti-GS (1:100, Santa Cruz), rabbit anti-GS (1:250, Proteintech), goat anti-guinea pig IgG (H + L) Alexa Fluor 647 (1:1,000, Thermo Fisher Scientific, Waltham, USA), goat anti-rabbit IgG (H + L) Alex Fluor 488 (1:1,000, Thermo Fisher Scientific), goat anti-chicken IgY (H + L) Alexa Fluor Plus 647 highly cross absorbed (Thermo Fisher Scientific), donkey anti-rabbit IgG (H + L) Alexa Fluor 647 (1:1,000, Thermo Fisher Scientific), and Cy3 donkey anti-rat IgG (H + L) (1:2,000; Jackson ImmunoResearch). To control for unspecific binding of the secondary antibody, negative controls were performed in which primary antibodies had been omitted. Finally, 4,6-diamidino-2-phenylindole (DAPI, Vector Laboratories) was added to counterstain nuclear DNA. Specimens were analyzed using a Zeiss Axio Imager microscope (Carl Zeiss AG, Jena, Germany).

### Expression analysis

Mice were anesthetized with CO_2_ and euthanized by atlanto-occipital dislocation. Eyes were enucleated and dissected to obtain the sensory retinal tissue. Total RNA was extracted with TriFast (Peqlab, Erlangen, Germany) according to the manufacturer’s recommendations. In brief, tissue was transferred to a Lysing Matrix D Tube (MP Biomedicals, Eschwege, Germany) containing 500 μL TriFast and homogenized in a FastPrep-24 (MP Biomedicals). Samples were centrifuged and transferred to a new tube. Next, 200 μL chloroform was added, vortexed for 15 s, and centrifuged for 20 min at 12,000 × *g* at 4°C to induce phase separation. The aqueous phase containing the total RNA was transferred into a new tube, precipitated with 100% isopropanol, washed twice with 75% ethanol, and dissolved in RNase-free water. cDNA synthesis from total RNA was performed using qScript cDNA Synthesis Kit (Quanta Biosciences, Gaithersburg, United States) according to the manufacturer’s instructions. Real-time RT-PCR was performed on a Bio-Rad CFXConnect Real-time System (Bio-Rad, Munich, Germany) with a temperature profile as follows: 95°C for 3 min, followed by 40 cycles of 10 s at 95°C, 20 s at 60°C, and 30 s at 72°C. All primers were purchased from Sigma-Aldrich (St. Louis, Missouri, USA) and extended over exon-intron boundaries: (*mmVegfa164* forward) 5′- ggagagatgagcttcctacagca-3′; (*mmVegfa164* reverse) 5′-ccttggcttgtcacatttttct-3′; (*mmAng-2* forward) 5′-cacactgaccttccccaact-3′; (*mmAng-2* reverse) 5′-cccacgtccatgtcacagta-3′; (*mmRpl32* forward) 5′-gctgccatctgttttacgg-3′; (*mmRpl32* reverse) 5′-tgactggtgcctgatgaact-3′. RNA that was not reverse transcribed served as the negative control. For relative quantification of the experiments, Rpl32 was used as a housekeeping gene. Bio-Rad CFX Software was used for analysis, and the ΔΔct method was applied for normalization.

### ELISA measurements

For quantification of huNorrin concentration in the retinae after AAV-mediated norrin expression, ELISA measurements were performed. Mice were anesthetized with CO_2_ and euthanized by atlanto-occipital dislocation. Eyes were enucleated and dissected to obtain the sensory retinal tissue. Tissue was transferred to a Lysing Matrix D Tube (MP Biomedicals, Eschwege, Germany) containing 200 μL RIPA buffer and homogenized in a FastPrep-24 (MP Biomedicals). Measurement of huNorrin concentration was performed using the Human NORN ELISA Kit (RayBio, USA) according to the manufacturer’s instructions.

### Transmission electron microscopy

For TEM, eyes were enucleated and fixed in Karnovsky’s solution (2.5% glutaraldehyde and 2.5% PFA in 0.1 M cacodylate buffer) for 24 h.^53^ After rinsing in 0.1 M cacodylate buffer, postfixation was accomplished in a mixture of 1% OsO_4_ and 0.8% potassium ferrocyanide in 0.1 M cacodylate buffer for 2 h at 48°C. After dehydration in a graded series of ethanol, the eyes were embedded in Epon (Serva, Heidelberg, Germany). Ultrathin sections were mounted on uncoated copper grids, stained with uranyl acetate and lead citrate, and examined on a Zeiss Libra transmission electron microscope (Carl Zeiss AG).

### Quantification fluorescence images

For quantification of retinal blood vessel percentage, eight images of the deep vascular plexus, two in each quadrant of the retinal flat mount, were obtained from each experimental sample. Using the software AngioTool (64.0.6a), the percentage of the deep vascular area relative to the total explant area was obtained. The mean of the twelve samples was calculated and analyzed. For the analysis of the immunohistochemical staining, the software ImageJ was used. The obtain exclusively the immunoreactivity in the retinal blood vessel, a mask was generated using the fluorescence signal of the fluorescein isothiocyanate (FITC) dextran, and with this mask, the signal of caveolin-1 and claudin-5 was calculated. The analysis of the avascular area, the area of neovascular tufts, and the area of the deep vascular plexus were performed by using ZEN (Carl Zeiss). Here, the area of these was quantified and calculated relative to the total retinal flat mount area. All quantitative measurements were performed on blinded samples.

### Functional ERG analysis

Mice were dark adapted for at least 12 h before experiments and anesthetized by subcutaneous injection of ketamine (65 mg/kg) and xylazine (13 mg/kg). Pupils were dilated with tropicamide eye drops (Mydriaticum Stulln; Pharma Stulln). Silver needle electrodes served as a reference (forehead) and a ground (tail), and gold wire ring electrodes served as active electrodes. Corneregel (Bausch & Lomb) was applied to keep the eye hydrated and to maintain good electrical contact. ERGs were recorded from both eyes simultaneously using a Ganzfeld bowl (Ganzfeld QC450 SCX; Roland Consult) and an amplifier and recording unit (RETI-Port; Roland Consult). Responses were band-pass filtered (1–300 Hz) and averaged. Single-flash scotopic (dark-adapted) responses to a series of 10 LED flash intensities ranging from −3.5 to 1.0 log cd.s/m^2^ with an interstimulus interval of 2–20 s for the highest intensity were recorded. After 10 min of adaptation to white background illumination (25 cd/m^2^), single-flash photopic (light-adapted) responses to four Xenon flash intensities (0.5–2 log cd.s/m^2^) were recorded. All analysis and plotting were performed with R 4.4.2 (The R Foundation for Statistical Computing) and ggplot2 3.5.2.^54^

### Data analysis and statistics

All experiments were repeated at least three times. Results are depicted as mean (SD). Student’s *t* test or one-way ANOVA were performed to determine statistical significance of data. Statistical analyses were performed using GraphPad Prism 9.5.1 (GraphPad Software, La Jolla, CA) with *p* ≤ 0.05 taken as statistically significant.

## Data and code availability

The raw data supporting the conclusion of this article will be made available by the corresponding author, without undue reservations.

## Acknowledgments

This work was supported by a research collaboration agreement between the 10.13039/501100005626University of Regensburg and 10.13039/100008349Boehringer Ingelheim Pharma GmbH & Co. KG. AAV vector constructs were provided by Boehringer Ingelheim Pharma GmbH & Co. KG. The sponsor had no role in experimental procedure, data collection, analysis and interpretation, decision to publish, or preparation of the manuscript.

## Author contributions

Conceptualization, A.E.D., H.F., B.S., N.R., and E.R.T.; data curation, A.E.D. and H.J.; formal analysis, A.E.D. and H.J.; investigation, A.E.D. and H.J.; methodology, A.E.D. and E.R.T.; project administration, E.R.T.; supervision, E.R.T.; visualization, A.E.D.; writing – original draft, A.E.D., H.J., and E.R.T.; writing – review and editing, A.E.D., H.J., H.F., B.S., N.R., and E.R.T.

## Declaration of interests

H.F. and B.S. are employees of Boehringer Ingelheim Pharma GmbH & Co. KG. N.R. is a retired employee of Boehringer Ingelheim Pharma GmbH & Co. KG.

## Supplemental information

Document S1. Figures S1–S3

Document S2. Article plus supplemental information
