# Supplementary material for: AAV-norrin gene therapy rescues retinal defects in mice with Norrie disease and oxygen-induced retinopathy
Source: Mol Ther Adv. 2026 Jun 24;34(3):201795. doi: 10.1016/j.omta.2026.201795 (PMC13355717; doi:10.1016/j.omta.2026.201795)
Supplement: Document S1. Figures S1–S3 [file mmc1.pdf]

OMTA, Volume *34*

Supplemental information

AAV-norrin gene therapy rescues retinal defects in mice with Norrie disease and oxygen-induced retinopathy

Andrea E. Dillinger, Herbert Jaegle, Holger Fuchs, Benjamin Strobel, Norbert Redemann, and Ernst R. Tamm

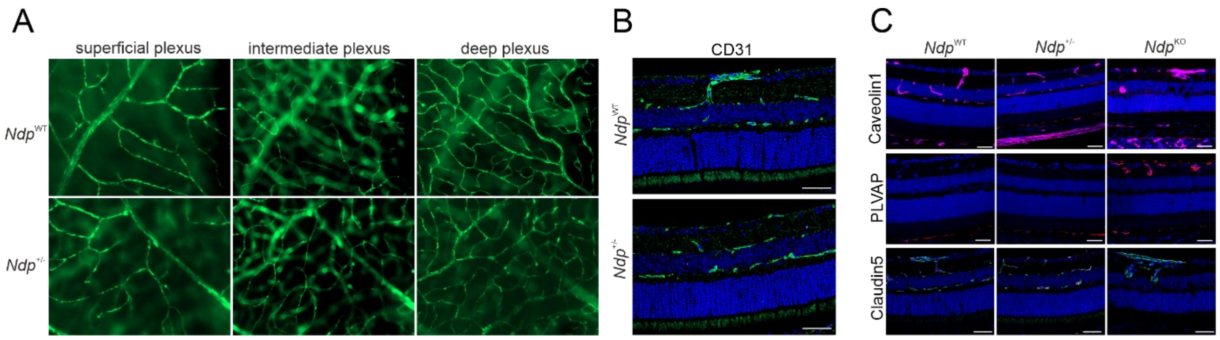

Figure S1: Analysis of retinal capillaries and blood-retinal barrier in *Ndp*^{WT} and *Ndp*$^{+/-}$ mice. (A) Exemplary images of the three capillary plexus (superficial, intermediate, deep) of FITC-Dextran (green) perfused retinal flat mounts of a *Ndp*^{WT} and *Ndp*$^{+/-}$ mouse. **(B)** FITC-Dextran (green) perfused retinal cross sections of *Ndp*^{WT} and *Ndp*$^{+/-}$ mouse. Nuclei were stained with Dapi (blue).. **(C)** Immunohistochemical staining of Caveolin-1 (magenta), PLVAP (red) and Claudin5 (magenta)on retinal cross sections of a *Ndp*^{WT}, *Ndp*$^{+/-}$ and *Ndp*^{KO} mouse. Nuclei were stained with Dapi.

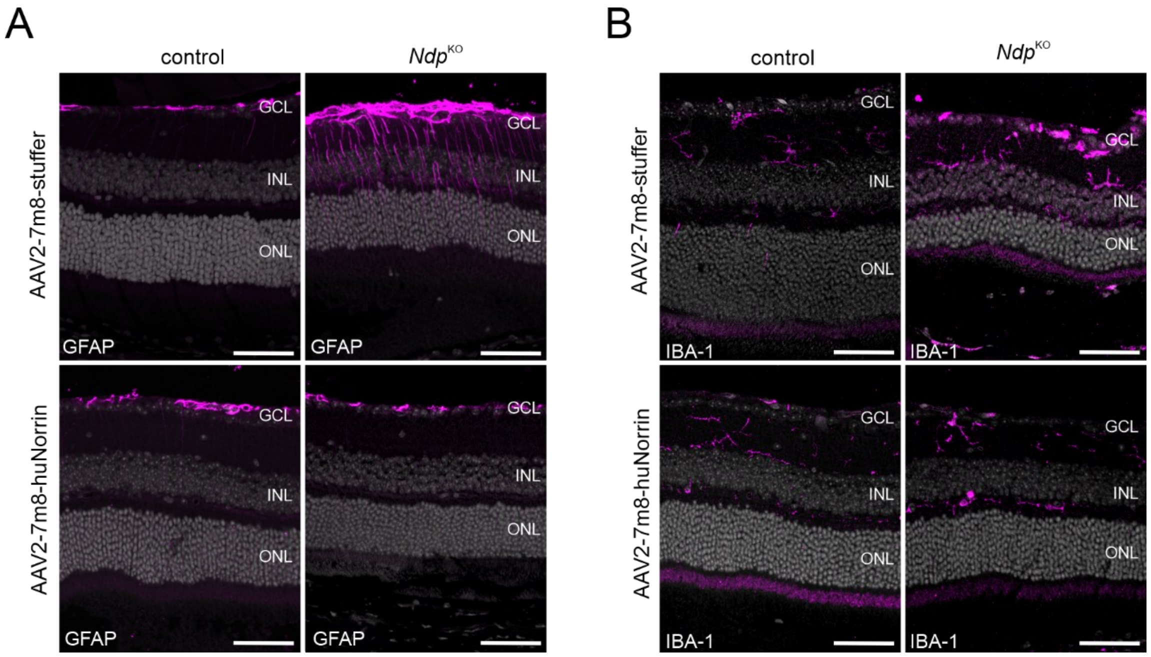

Figure S2: AAV-mediated norrin expression reduces micro-and macroglial activation in Norrin deficient mice. (A) Immunohistochemical staining of GFAP (magenta) in retinal gross sections (green) from control and *Ndp*^{KO} mice after intravitreal injection of AAV2-7m8-stuffer or AAV2-7m8-huNorrin. Nuclei, Dapi (gray). Scale bar: 50µm. (B) Immunohistochemical staining of IBA-1 (magenta) in retinal gross sections (green) from control and *Ndp*^{KO} mice after intravitreal injection of AAV2-7m8-stuffer or

AAV2-7m8-huNorrin. Nuclei, Dapi (gray). Scale bar: 50µm

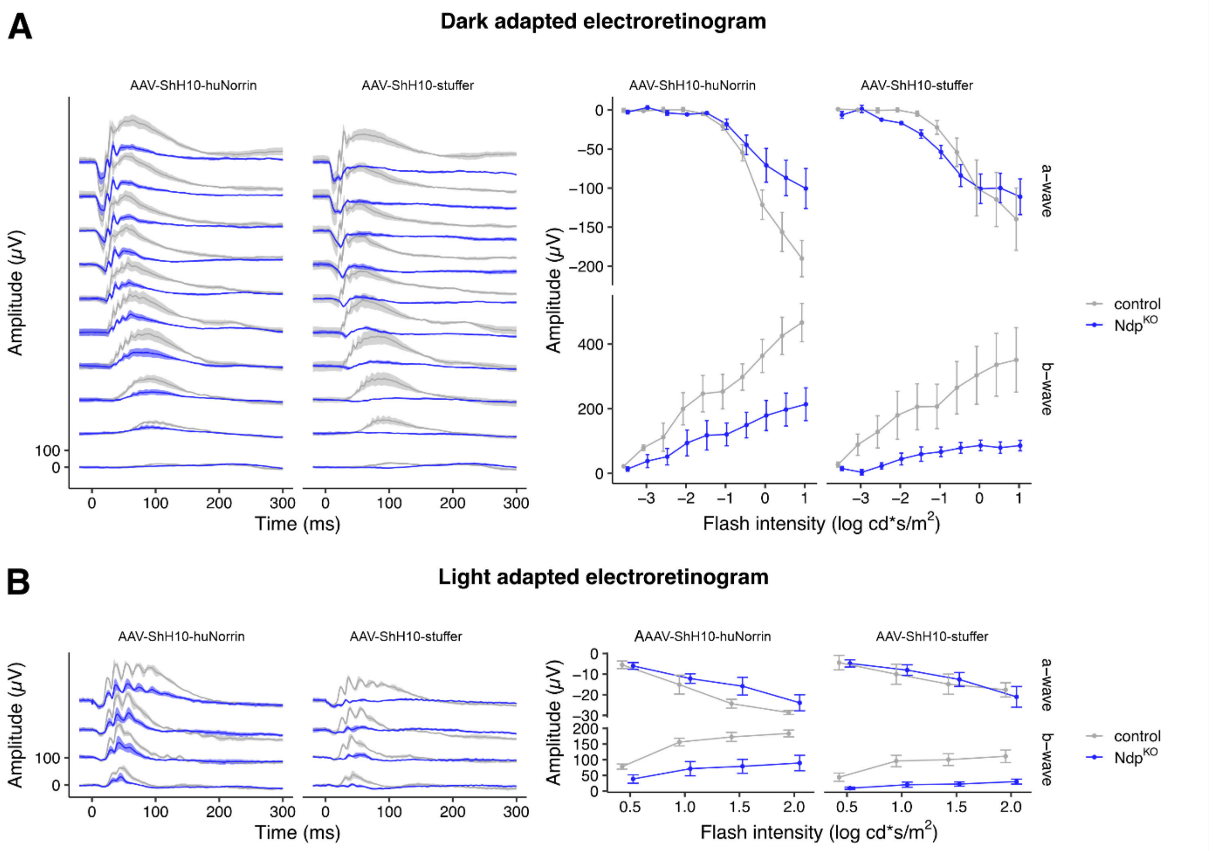

Figure S3: AAV-ShH10-mediated norrin expression restores visual function in *Ndp*-deficient mice. (A, B). Dark-adapted rod (A) and light-adapted cone ERG (B) waveforms and peak amplitudes of *in vivo* electroretinogram recordings of control and *Ndp*^{KO} mice after intravitreal injection of AAV-ShH10-stuffer or AAV-ShH10-huNorrin.
